# Ultrasound‐Assisted Soaking and Solution Effects on the Anti‐Nutritional Quality and Physical Properties of Legume Seeds

**DOI:** 10.1002/fsn3.70152

**Published:** 2025-04-06

**Authors:** Bekir Gökçen Mazı, Kübra Çağlayan

**Affiliations:** ^1^ Department of Food Engineering, Agricultural Faculty Ordu University Turkey

**Keywords:** anti‐nutritional factor, hardness, legume seed, soaking, solution acidity, ultrasound

## Abstract

This study investigates the effects of soaking method (ultrasound‐assisted soaking (UAS) and conventional soaking (CS)), soaking solutions with varying pH (distilled water (W), citric acid (CA), and sodium bicarbonate (SB)), and soaking times (4, 8, and 12 h) on anti‐nutritional factors (phytic acid (PA) and trypsin inhibitor (TI) contents), moisture absorption, and hardness of chickpeas, beans, and soybeans. Additionally, the study examined changes in soluble solids content (SSC), pH, turbidity, and color of the soaking solutions. While the soaking method and solution significantly (*p* < 0.05) affected TI content in seeds, they had no notable impact on PA content. The most notable reductions in PA were observed following 12 h of CS: 27.0% in chickpeas and 38.9% in soybeans soaked in water, and 30.5% in beans soaked in sodium bicarbonate. Sodium bicarbonate was more effective than citric acid and water in reducing TI. UAS generally led to greater TI reductions compared to CS, although its effectiveness was reduced in sodium bicarbonate. UAS resulted in higher hydration rates across all solutions. Chickpeas and beans soaked in citric acid for 4 h and soybeans soaked in citric acid for 12 h exhibited the lowest hardness values. UAS notably increased SSC, turbidity, and pH changes in the soaking solutions compared to CS.

## Graphical abstract

This study investigates the effects of soaking method (ultrasound‐assisted soaking and conventional soaking), soaking solutions with varying pH (distilled water, citric acid, and sodium bicarbonate), and soaking times (4, 8, and 12 h) on anti‐nutritional factors (phytic acid and trypsin inhibitor contents), moisture absorption, and hardness of chickpeas, beans, and soybeans. Additionally, the study examines changes in soluble solids content, pH, turbidity, and color of the soaking solutions.

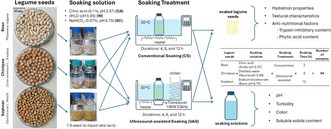

## 1 Introduction

Edible legumes, the seeds of the family Leguminosae, are an important source of affordable and high‐quality protein. However, legumes can also contain various anti‐nutritional factors such as enzyme inhibitors, lectins, tannins, phytic acid (PA), oligosaccharides, and saponins, which can have potential adverse physiological and functional effects (Nasab et al. 2024; Nath et al. 2022; Muzquiz et al. 2012). Among these, PA and trypsin inhibitor (TI) are particularly relevant due to their impact on nutrient bioavailability and protein digestibility, making them key targets for reduction through processing techniques. PA, also known as myo‐inositol 1,2,3,4,5,6‐hexakisphosphoric acid, is the primary phosphorus reserve in cereals and legumes. PA is commonly referred to as an anti‐nutrient because it negatively affects the absorption of multivalent cations such as calcium, zinc, magnesium, and iron in the gastrointestinal system by forming complexes with these positively charged ions (Zhang et al. 2022). Trypsin inhibitors (TIs), a type of serine protease inhibitors, can disrupt protein digestion and reduce protein bioavailability by inhibiting pancreatic enzyme trypsin (Muzquiz et al. 2012). Additionally, since many sulfur‐containing amino acids in legume seed proteins are part of the structure of protease inhibitors, a sulfur deficiency can occur in diets based on legume seeds (Muzquiz et al. 2012). On the other hand, these compounds have also been linked to health‐promoting properties (Feizollahi et al. 2021; Nath et al. 2022). The anti‐nutritional properties of PA or protease inhibitors can lead to certain health problems, particularly in developing countries where diets are of poor quality and heavily reliant on cereals and legumes as staple foods. Therefore, to minimize the negative effects of these compounds on long‐term health, their levels in the diet need to be reduced.

Various traditional and novel food processing techniques, such as soaking, cooking, germination, extrusion, dehulling, fermentation, irradiation, pulsed electric field, and ultrasound, have been investigated for their effectiveness in reducing anti‐nutritional factors (Nasab et al. 2024; Kheto et al. 2024b; Feizollahi et al. 2021). Soaking is a common pre‐processing step to prepare legumes for subsequent processing steps such as cooking, canning, or fermentation. Proper hydration during soaking ensures that legumes cook uniformly in a shorter time. Soaking also helps the removal of anti‐nutritional compounds (Feizollahi et al. 2021). Soaking efficiency can be improved by changing the process parameters. It has been shown in various studies that soaking time (ST) and acidity of the soaking medium are important factors influencing the hydration level and reduction of anti‐nutritional factors (Vidal‐Valverde et al. 1997; Frias et al. 2000; Haladjian et al. 2003; Huma et al. 2008; Vasquez et al. 2021; Feizollahi et al. 2021). The findings of these studies reveal that the effect of the soaking solution acidity on hydration rate and anti‐nutritional components varies depending on the type of legume seed and processing factors.

Since soaking is a time‐consuming batch‐wise process, accelerating the process is crucial. High temperature during soaking is a commonly used method to shorten the ST. However, higher temperatures may result in higher loss of water‐soluble substances (Devkota et al. 2022). Therefore, various studies in the literature have explored different methods to accelerate soaking other than increasing temperature. Among these, ultrasound‐assisted soaking (UAS) has gained attention for its ability to enhance hydration efficiency while preserving nutritional quality (Kheto et al. 2024a; Nasab et al. 2024; Patero and Augusto 2015; Miano et al. 2019; Vasquez et al. 2021; Kumar et al. 2023). The use of ultrasound during soaking affected the color and texture characteristics of the chickpeas, as well as the properties of the soaking water (Yildirim et al. 2013; Yıldırım and Öner 2015). Han and Baik (2006) noted that soaking with ultrasound may promote leaching of oligosaccharides to soaking water. Upon reviewing the conducted studies, it is evident that research focusing on the effects of UAS on the anti‐nutritional factors and physical properties of legume seeds, as well as on the characteristics of the soaking medium, remains limited.

The primary objective of this study is to investigate the effects of acidity of soaking solutions (distilled water, 0.1% citric acid solution, and 0.07% sodium bicarbonate solution) during UAS and conventional soaking (CS) over varying STs (4, 8, and 12 h) on the levels of PA and TI in chickpeas, soybeans, and beans. Additionally, the study aims to evaluate the impact of these processing parameters on the moisture content and hardness of the legume seeds, as well as the properties of the soaking solutions.

## 2 Materials and Methods

### 2.1 Materials

**FIGURE 1:**
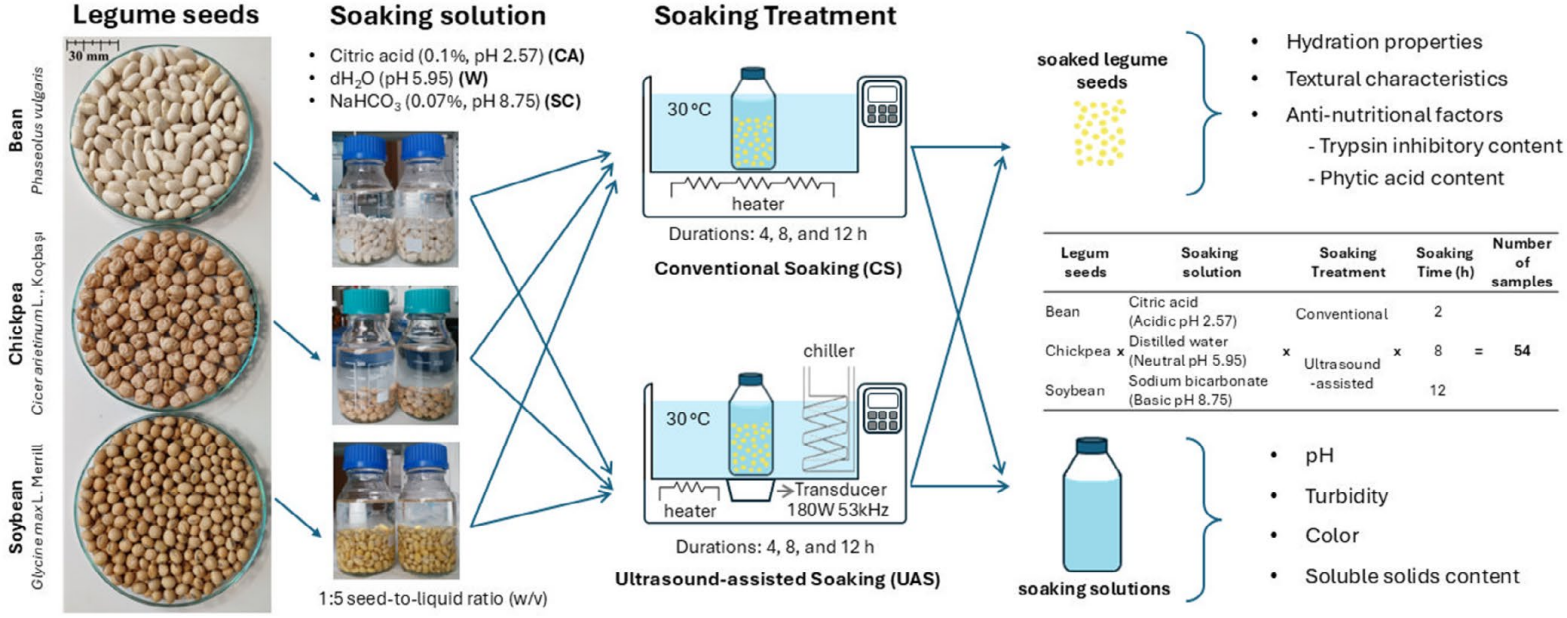
Schematic representation of experimental methodology.

Bean ( *Phaseolus vulgaris* ), chickpea ( *Cicer arietinum* L., Koçbaşı), and soybean ( *Glycine max* L. Merrill) seeds were purchased from a local market in Türkiye (Figure 1). All chemicals were obtained from Merck (Darmstadt, Germany) and Sigma Chemicals Co. (St. Louis, United States) and were of analytical grade.

### 2.2 Soaking Treatments

CS and UAS methods were employed for chickpeas, beans, and soybeans. The flowchart of soaking treatments was given in Figure 1. Utilizing the CS technique, the process was conducted within a water bath (Wsb‐18, WiseBath, Daihan Scientific, South Korean), while in the UAS, it was carried out in an ultrasonic water bath (53 kHz, 180 W, amplitude level 100%) (SK3310HP, Kudos, China). After removing the degraded seeds and impurities, the legumes (50 g) were placed in glass jars containing soaking solution (250 mL). The soaking process was conducted with a constant seed‐to‐soaking solution ratio of 1:5 (w/v) (Bishnoi et al. 1994; Alonso et al. 2000) and a fixed soaking temperature of 30°C (Bishnoi et al. 1994; Alonso et al. 2000; Ulloa et al. 2015; Adeleke et al. 2017; López López et al. 2017), as established in previous studies. The treatment variables included different STs (2, 4, and 8 h) and various soaking solutions (acidic (0.1% citric acid), neutral (distilled water), and basic (0.07% sodium bicarbonate)), as referenced in the literature (Vidal‐Valverde et al. 1994; Frias et al. 2000; Prodanov et al. 2004). A modular cooling serpentine with a refrigerated circulating bath (ADO7R‐20‐A12E; PolyScience, Niles, Illinois, USA) was used to maintain a constant temperature (30°C) during UAS. After soaking, the legume seeds were removed from the soaking medium, strained, and blotted with absorbent paper to remove surface moisture.

### 2.3 Moisture Content of Seeds

Moisture content of raw and soaked legumes was determined by drying until constant weight at 105°C using a moisture analyzer (Radwag, MAC 50, Poland) (Han et al. 2010). Average moisture content was reported on a percentage wet basis (w.b.). Raw and soaked whole legumes were ground (PRG 259, Premier, Istanbul, Türkiye) before measurements.

### 2.4 Hardness of Seeds

The textural characteristics of the soaked legume samples were obtained by a texture analyzing instrument (Texture Analyzer TA.XT.plus (Stable Micro System, England)) equipped with a 36 mm (P/36R) cylindrical probe at room temperature. Analysis was carried out by placing the legume seed centered on the plate part of the texture analyzer. The settings used were: pre‐test speed: 1.0 mm/s; test speed: 1.0 mm/s; post‐test speed: 1.0 mm/s; Strain: 75% (Singh et al. 2010). Values of hardness were calculated by using instrument software (Texture Exponent v.6.1.1.0, Stable Microsystems). For each sample, results represent the arithmetic mean of eight replications.

### 2.5 Anti‐Nutritional Factors of Seeds

Trypsin inhibitory content (TIC) in raw and soaked legume seed was determined using the spectrophotometric method (UV 1240, Shimadzu, Kyoto, Japan) at a wavelength of 412 nm, with Nα‐benzoyl‐DL‐arginine‐p‐nitroanilide hydrochloride (BAPNA) as the substrate (Luo and Xie 2013). One gram of the sample was mixed with 100 mL of 0.009 M HCl at room temperature. After shaking with a reciprocating water bath (WSB 18L, Daihan Scientific, Korea) at 25°C and 50 RPM for 1 h, it was centrifuged (Micro 220‐R, Hettich‐Zentrifugen, Germany) at 25°C and 10,000 *g* for 20 min. The clear supernatant that resulted was used to measure the TIC. Trypsin solution was prepared by dissolving 4 mg of accurately weighed trypsin (Sigma‐Aldrich T4799, from porcine pancreas) in 200 mL of 1 mM HCl with 5 mM CaCl_2_. Substrate solution was prepared by dissolving 40 mg of BAPNA (Sigma‐Aldrich B3133) in 1 mL of dimethyl sulfoxide (DMSO, Sigma‐Aldrich D2650) and diluted to 100 mL tris‐buffer (50 mM Tris–HCl, pH 8.2, and 5 mM CaCl2) prewarmed to 37°C (Kakade et al. 1974). The assay was conducted by combining 0.12 mL of diluted sample extract with 0.3 mL of BAPNA solution. Following a 10 min incubation period at 37°C, 0.3 mL of trypsin solution was introduced, and the sample was subsequently incubated for an additional 10 min at 37°C. The reaction was terminated by the addition of 0.06 mL of 30% acetic acid. Using an equation developed by Hamerstrand et al. (1981), TIC was determined from absorbance measurements. The findings are expressed as milligrams of trypsin inhibitor per gram of sample.

$$\mathrm{TIC}=\frac{A_{\mathrm{std}}-A_{\text{sample}}}{0.019\times \text{sample}\;\mathrm{wt}.\left(g\right)}\times \frac{\text{dilution factor}}{1000\times \text{sample volume}\;\left(\mathrm{mL}\right)}$$

The determination of PA content (PAC) in raw and soaked legume seed was carried out using the colorimetric method (Haug and Lantzsch 1983). This method involved the extraction of PA from the sample using 0.2 M hydrochloric acid solution. Following this, the extract was treated with a Ferric solution containing 0.2 g of ammonium iron (III) sulfate 12 H_2_O (Merck Art. 103776) dissolved in 100 mL of HCl and made up to 1000 mL with distilled water. The iron bound to PA was then precipitated, and the amount of iron remaining in the supernatant (separated by centrifugation at 4000 *g* for 30 min using the Universal 320‐R centrifuge from Hettich‐Zentrifugen, Germany) was determined spectrophotometrically at 519 nm. Results were reported in mg of PA per 100 g of sample.

### 2.6 Analysis of Soaking Solution Properties

The measurements of the pH levels were carried out using a pH meter (Starter 3100, Ohaus, MA, USA) (Mazı et al. 2023). The digital handheld refractometer (DR201‐95, Kruss Optronic, Hamburg, Germany) was utilized to determine the soluble solids content (g/100 mL) of the soaking water samples. The turbidity of the soaking water samples was evaluated using the spectrophotometric method at a wavelength of 500 nm (Bayram et al. 2004). The measurements were performed at room temperature, with distilled water serving as a blank. Color measurements of the soaking solutions were obtained using a color measurement device in conjunction with a quartz cuvette (D: 63.5 mm, h: 38.2 mm). The data obtained for each sample (50 mL) represent the average of five separate replications.

### 2.7 Statistical Analysis

The data were assessed using a one‐way and/or three‐way analysis of variance (ANOVA). Differences among individual means were compared by using Tukey Comparison test (*p* < 0.05). Pearson correlation coefficients for the properties of chickpea, bean, and soybean seeds were calculated to determine the relationships between variables. Additionally, Principal Component Analysis (PCA) was performed as a multivariate analysis to identify patterns within the dataset. All statistical analyses were conducted using Minitab (Version 17).

## 3 Results and Discussion

### 3.1 PA Content

Legume seeds contain PA (myo‐inositol‐(1,2,3,4,5,6) hexakisphosphate) and its salt form (phytate) at varying levels (Muzquiz et al. 2012). In this study, PA content of raw chickpea, bean, and soybean was measured as 1064, 1385, and 1834 mg/100 g, respectively. These values fall within the ranges reported in the literature (Zhang et al. 2022; Shi et al. 2018). Among the tested legumes, soybean exhibited the highest PA content. Following soaking treatments, the PA content decreased to a range of 777–1062 mg/100 g for chickpeas (representing a 0%–27% reduction), 962–1361 mg/100 g for beans (a 2%–31% reduction), and 1121–1800 mg/100 g for soybeans (a 1%–39% reduction) (Table 1). The reduction in PA content during soaking occurs primarily through two mechanisms: the leaching of soluble phytate salts into the soaking medium and the enzymatic hydrolysis of phytate by endogenous phytase (Feizollahi et al. 2021). Samia et al. (2013) reported losses of 24.8% and 27.8% in the PA contents of chickpea and faba bean, respectively, after soaking in distilled water for 12 h at room temperature. In a different study, Shi et al. (2018) observed that soaking whole chickpea, soybean, and common beans in distilled water for 4 h at room temperature led to reductions of 0.32%, 2.82%, and 0.54%–2.34%, respectively, in PA content. The reductions achieved in our study during CS treatments are comparable to these results.

**TABLE 1:** Anti‐nutritional factors of legume seeds under conventional soaking (CS) and ultrasound‐assisted soaking (UAS).

Soaking treatment	Soaking time (h)	Chickpea		Bean		Soybean	
		Trypsin inhibitors (mg/g)	Phytic acid (mg/100 g)	Trypsin inhibitors (mg/g)	Phytic acid (mg/100 g)	Trypsin inhibitors (mg/g)	Phytic acid (mg/100 g)
Raw		16.04 ± 0.34	1064 ± 37	22.44 ± 0.43	1385 ± 35	58.55 ± 0.28	1834 ± 47
CS‐W	4	12.38 ± 0.18a*A** (22.8)	1062 ± 37aA (0.2)	13.76 ± 0.14aA (37.6)	1361 ± 35aA (1.7)	44.52 ± 0.18aA (24.0)	1694 ± 193aA (7.6)
	8	12.09 ± 0.16aA (24.6)	920 ± 44abA (13.5)	13.44 ± 0.13aA (39.0)	1090 ± 10bA (21.3)	33.64 ± 0.11bA (42.5)	1343 ± 134aA (26.8)
	12	5.08 ± 0.11bB (68.3)	777 ± 65bA (27.0)	5.65 ± 0.09bB (74.4)	992 ± 13cA (28.4)	26.51 ± 0.09cB (54.7)	1121 ± 63aB (38.9)
CS‐CA	4	9.03 ± 0.07aB (43.7)	1022 ± 41aA (3.9)	10.04 ± 0.09aB (54.4)	1273 ± 113aA (8.1)	39.15 ± 0.07aC (33.1)	1815 ± 99aA (1.0)
	8	8.88 ± 0.06aB (44.6)	1013 ± 47aA (4.8)	9.87 ± 0.09aB (55.2)	1260 ± 181aA (9.0)	30.15 ± 0.10bAB (48.5)	1562 ± 110aA (14.8)
	12	5.93 ± 0.18bA (63.0)	855 ± 35aA (19.6)	6.59 ± 0.06bA (70.1)	1090 ± 45aA (21.3)	28.10 ± 0.06cA (52.0)	1507 ± 87aA (17.8)
CS‐SB	4	8.95 ± 0.09aBC (44.2)	1025 ± 34aA (3.7)	9.95 ± 0.03aB (54.9)	1263 ± 87aA (8.8)	35.03 ± 0.03aD (40.2)	1695 ± 102aA (7.6)
	8	6.17 ± 0.11bD (61.5)	936 ± 59abA (12.0)	6.86 ± 0.09bD (68.9)	1036 ± 55abA (25.2)	29.09 ± 0.14bAB (50.3)	1480 ± 20abA (19.3)
	12	3.78 ± 0.01cD (76.4)	836 ± 28bA (21.4)	4.20 ± 0.16cD (80.9)	962 ± 39bA (30.5)	12.69 ± 0.16cF (78.3)	1373 ± 72bAB (25.1)
UAS‐W	4	8.41 ± 0.06aC (47.6)	1010 ± 41aA (5.1)	9.35 ± 0.07aC (57.6)	1149 ± 16aA (17.0)	28.74 ± 0.07aE (50.9)	1575 ± 154aA (14.1)
	8	7.07 ± 0.16bC (55.9)	1030 ± 111aA (3.2)	7.85 ± 0.27bC (64.4)	1058 ± 48aA (23.6)	25.78 ± 0.27bAB (56.0)	1451 ± 171aA (20.9)
	12	5.82 ± 0.04cA (63.7)	907 ± 97aA (14.8)	6.47 ± 0.11cA (70.6)	1029 ± 139aA (25.7)	14.85 ± 0.16cC (74.6)	1316 ± 98aAB (28.2)
UAS‐CA	4	6.91 ± 0.27aD (56.9)	994 ± 16aA (6.6)	7.69 ± 0.18aD (65.1)	1244 ± 30aA (10.2)	40.96 ± 0.28aB (30.0)	1629 ± 168aA (11.2)
	8	5.85 ± 0.16bDE (63.5)	971 ± 27abA (8.7)	6.50 ± 0.09bDE (70.5)	1082 ± 26aA (21.9)	24.00 ± 0.14bB (59.0)	1440 ± 188aA (21.5)
	12	4.67 ± 0.13cBC (70.9)	832 ± 58bA (21.8)	5.20 ± 0.09cC (76.4)	1048 ± 168aA (24.3)	14.02 ± 0.09cD (76.1)	1437 ± 158aAB (21.6)
UAS‐SB	4	8.81 ± 0.11aBC (45.1)	1004 ± 54aA (5.6)	9.80 ± 0.26aBC (55.5)	1247 ± 67aA (10.0)	29.19 ± 0.18aE (50.1)	1800 ± 45aA (1.9)
	8	5.49 ± 0.09bE (65.8)	932 ± 60aA (12.4)	6.10 ± 0.13bE (72.3)	1052 ± 80bA (24.0)	23.40 ± 0.06bB (60.0)	1588 ± 141aA (13.4)
	12	4.50 ± 0.24cC (71.9)	861 ± 143aA (19.1)	5.00 ± 0.04cC (77.3)	1070 ± 35abA (22.7)	13.46 ± 0.06cE (77.0)	1466 ± 41aAB (20.1)
**Source**		***p* (*R*** ^**2**^ **= 0.99)**	***p* (*R*** ^**2**^ **= 0.77)**	***p* (*R*** ^**2**^ **= 0.99)**	***p* (*R*** ^**2**^ **= 0.76)**	***p* (*R*** ^**2**^ **= 0.99)**	***p* (*R*** ^**2**^ **= 0.79)**
*SM*		0.000***	0.630	0.000	0.209	0.000	0.770
*SS*		0.000	0.743	0.000	0.218	0.000	0.012
*ST*		0.000	0.000	0.000	0.000	0.000	0.000
*SM × SS*		0.000	0.213	0.000	0.237	0.000	0.083
*SM × ST*		0.000	0.325	0.000	0.239	0.042	0.391
*SS × ST*		0.000	0.808	0.000	0.709	0.001	0.706
*SM × SS × ST*		0.000	0.593	0.000	0.000	0.000	0.761

*Note:* Values are means ± standard deviation of two replicates. Values in parentheses indicate % decrease over raw values. *For each soaking treatment, different small case letters in the same column indicates significant difference between soaking times (*P* < 0.05). **For each soaking time, different capital letters in the same column indicates significant difference between soaking treatments (*p* < 0.05). ***p < 0.05 denotes significant effect of main factors or interactions.

Abbreviations: CA, citric acid; SB, sodium bicarbonate; SM, soaking method; SS, soaking solution; ST, soaking time; W, water.

ST was identified as a significant factor (*p* < 0.05) influencing the PA content of all seeds. The PA content of the seeds decreased with increasing ST, with a more pronounced effect observed during CS compared to UAS. Extending the ST from 4 to 12 h resulted in a reduction of 14%–34% in PA content during CS treatments, while it led to a reduction of 10%–19% during UAS treatments. Prolonged soaking increased cumulative PA loss through the combined effects of leaching and enzymatic hydrolysis. However, the rate of PA reduction did not remain consistent with increasing ST; instead, it varied depending on the seed type and the soaking solution used. Changes in the pH of the soaking medium may influence the rate of PA loss, as both phytase enzyme activity and the solubility of phytate salts are pH‐dependent (Wang and Guo 2021). The initial pH values of distilled water, citric acid, and sodium bicarbonate used in this study were measured as 5.95, 2.57, and 8.75, respectively. Most plant phytases typically demonstrate optimal activity around pH 5.0–5.5; additionally, legumes may display phytate‐degrading activity at pH 8.0 (Greiner and Konietzny 2006). After 4‐h CS of chickpeas and beans, there was an increase in the pH of the citric acid solution (pH 3.7–3.9) and distilled water (pH 6.2–6.3) and a reduction in that of sodium bicarbonate solution (pH 7.1). The low pH (< 3.9) of citric acid solutions restricts the enzymatic hydrolysis of phytate. On the other hand, these acidic conditions (pH < 4) enhance the solubility of phytate (Amat et al. 2023; Crea et al. 2008). This suggests that, during 4‐h CS in citric acid, the primary loss of PA may have occurred via leaching. At this soaking condition, citric acid provided 3.8% and 6.5% lower PA content in chickpeas and beans, respectively, compared to distilled water, while yielding values similar to those observed with sodium bicarbonate. Our finding aligns with the finding of Mohammadi et al. (2021) who reported that 1 h‐soaking of rice bran at an acidic pH (pH = 2) resulted in greater PA reduction compared to soaking under neutral conditions (pH = 6). PA forms complexes with various mineral cations due to its high density of negatively charged phosphate groups. The solubility of these complexes is highly dependent on pH, type of cations, and cation to phytate ratio. In general, Ca and Mg salts of PA tend to be soluble at low pH and lower cation: phytate molar ratios (Pontoppidan et al. 2007; Crea et al. 2008; Cheryan and Rackis 1980). Cheryan et al. (1983) reported that below pH 5, Mg^2+^‐phytate complexes tend to remain soluble over a wide range of Mg^2+^:phytate ratios. However, during extended STs (8 and 12 h), citric acid (pH 4.1–4.6) resulted in 2%–22% higher PA content compared to distilled water (6.0–6.2) and sodium bicarbonate (pH 6.2–6.8) in chickpeas and beans. Similarly, CS of soybeans in citric acid (pH 4.3–4.7) caused 6%–34% higher PA content compared to water (pH 6.1–6.3) or sodium bicarbonate (pH 6.7–7.1). This may be related to the rise of pH above 4.0 in citric acid solution under these soaking conditions, which reduces the solubility of mineral‐phytate complexes and consequently limits further PA loss through leaching. Grynspan and Cheryan (1983) reported that in pure Ca‐phytate systems, as pH increases above 4, the solubility of phytate phosphorus decreases, with the extent of reduction depending on the Ca:PA molar ratio. However, phytate solubility is not solely governed by mineral interactions, as protein presence significantly influences its behavior. Phytate interacts with proteins through various binding mechanisms, forming binary phytate‐protein complexes that can further alter solubility (Pontoppidan et al. 2007). Phytate also forms ternary complexes with proteins and minerals (Amat et al. 2023). Phytate, with its multiple negatively charged phosphate groups, interacts strongly with positively charged proteins, especially at low pH (below pI of protein), leading to the formation of insoluble phytate‐protein complexes that can precipitate out of solution (Amat et al. 2023). However, in their study on phytate interactions with proteins and minerals, Pontoppidan et al. (2007) found that adding 5 g Ca^2+^ kg^−1^ significantly reduced phytate solubility at pH > 4.4, while at pH 1.5–4.4, Ca slightly increased the solubility of phytate. This increase at low pH is likely due to Ca competing with proteins for phytate binding, preventing phytate‐protein precipitation by forming soluble Ca‐phytate complexes. Grynspan and Cheryan (1989) investigated the solubilities of mixtures of phytate, Ca, and soy protein isolate and indicated that, from pI to pH 6.5, instead of phytate simply binding to Ca and precipitating as Ca‐phytate, a ternary complex (phytate‐Ca‐protein) may form. Therefore, phytate remains more soluble than expected until pH reaches 6.5, where a new critical pH triggers precipitation. All these interactions depend on various factors like pH, presence of cations, type and concentration of components, and molar ratio between components, which affect the extent and nature of complex formation, ultimately altering the solubility of PA (Amat et al. 2023). Given the complexity of interactions between phytate, proteins, and minerals, accurately predicting their impact on solubility remains challenging for this study. Overall, the effect of soaking solution (SS) on PA loss was found to be significant (*p* < 0.05) only for soybeans.

In UAS treatments, the influence of SS on PA content was less remarkable than that observed in CS treatments. The influence of ultrasound application on PA content varied depending on the type of seed, SS, and ST. In citric acid soaking treatments, UAS provided 2%–14% lower PA content in seeds compared to CS. This may be caused by the higher pH values achieved in citric acid solution during UAS, which may enhance the enzymatic hydrolysis of PA. In water soaking treatments, UAS gave 5%–16% lower PA values in seeds compared to CS following 4 h‐soaking, while it gave 4%–17% higher PA values following 12 h‐soaking. A similar trend was observed in chickpeas and beans but not in soybeans during sodium bicarbonate soaking treatments. Overall, across all soaking solutions, UAS yielded a slightly higher loss of PA following 4 h‐soaking treatments compared to CS. Similar results on phytate content reduction with ultrasound application have been reported by Yadav et al. (2021) for finger millet soaked in water for 30 min and by Mohammadi et al. (2021) for rice bran soaked in solutions of varying acidity for 1 h. In a different study, Kheto et al. (2024a) observed a reduction in PA content in guar seeds when ultrasound pretreatment was applied before soaking in water. The greater reduction in PA content observed during UAS can be at least partially attributed to the acoustic effects of cavitation, which disrupt surface structures, increasing surface area and enhancing the extraction rate of PA compounds into the soaking solution (Kheto et al. 2024a; Mohammadi et al. 2021). Additionally, ultrasound may facilitate the cleavage of molecular bonds in PA through cavitation‐induced effects (Kheto et al. 2024a; Nasab et al. 2024). Furthermore, several studies have demonstrated that ultrasound significantly affects the secondary and tertiary structures of proteins, primarily due to cavitation, which generates localized high shear forces, turbulence, and transient high temperatures and pressures (Kozell et al. 2023). These effects can disrupt hydrogen bonds and electrostatic interactions, leading to partial unfolding or denaturation of the proteins (Kozell et al. 2023; Qian et al. 2023). This structural modification may reduce protein affinity for PA, thereby increasing the solubility of PA in the soaking medium. At prolonged STs, it is not possible to make a clear conclusion regarding the effect of ultrasound application on PA loss. Statistically speaking, the soaking method (SM) was not determined to be a significant factor (*p* < 0.05) affecting the PA levels in chickpeas, beans, and soybeans. The highest level of PA loss occurred in water‐soaked chickpeas (27.0% reduction), water‐soaked soybeans (38.9% reduction) and sodium bicarbonate‐soaked beans (30.5% reduction) after 12 h of CS.

### 3.2 Trypsin Inhibitor (TI) Content

Trypsin inhibitor (TI) content of raw chickpea, bean, and soybean was 16.04, 22.04, and 58.55 mg/g, respectively. The highest TI content was found in soybean. Following soaking treatments, TI content decreased to a value between 3.78 and 12.38 mg/g for chickpeas, 4.20 and 13.76 mg/g for beans, and 12.69 and 44.52 mg/g for soybeans (Table 1). Reduction of TIs during CS and UAS treatments may be linked to the leaching of it into the soaking solution and disruption of protein structure. The main factors of SM, ST, soaking solution (SS), and the interactions of them were all found to be effective factors in TI contents of the seeds. It was observed that as the ST increased, the loss of TI also increased. During CS of chickpeas and beans in water and citric acid, extending the ST from 4 to 8 h resulted in a negligible (1.7%–2.3%) reduction in TI content, while extending the ST from 8 to 12 h led to a significant (33.2%–58.0%) reduction (*p* < 0.05). In all other samples subjected to CS and UAS, the reduction of TI content with increasing ST followed an almost linear pattern (*R* ^2^ ≥ 0.88). In general, the reduction trend in beans and chickpeas for both UAS and CS across all solutions followed a similar pattern, whereas soybeans exhibited a distinctly different reduction trend. This difference may be linked to the differences in the solubility properties, structural characteristics, and stability of TIs, present in these legume seeds. Legume TIs are classified into two major families based on their molecular size and structural properties: Kunitz trypsin inhibitors (KTIs) and Bowman‐Birk trypsin inhibitors (BBTIs). These two inhibitor types differ in their resistance to physical and chemical deactivation, influencing their behavior during soaking. The distribution of these inhibitors varies among legumes; for example, soybeans contain both KTIs and BBTIs, while common beans ( *Phaseolus vulgaris* ) and chickpeas ( *Cicer arietinum* ) primarily contain inhibitors from the Bowman‐Birk family (Norioka et al. 1988).

During CS, the effect of the SS on TI content varied with the SM, type of seed, and ST. For chickpeas and beans, the most notable distinction occurred between the samples soaked in sodium bicarbonate and water for 8 h, with water‐soaked samples exhibiting approximately twice the TI content compared to sodium bicarbonate‐soaked ones. Soybeans soaked in citric acid for 12 h were similarly found to have approximately twice the TI content compared to those soaked in sodium bicarbonate for a similar duration. In general, sodium bicarbonate led to a higher reduction in TI content in all legume seeds compared to water and citric acid during CS. Considering all samples, the highest reduction in TI content was obtained in chickpeas, beans, and soybeans subjected to CS in sodium bicarbonate solution for 12 h. This result is in good agreement with those reported by Vidal‐Valverde et al. (1997) in faba bean, by Shimelis and Rakshit (2007) in kidney bean, and by Marquez and Alonso (1999) in chickpea, but differs from that presented by Frias et al. (2000) who found that soaking chickpeas in distilled water (pH = 5.3) for 9 h caused a 12% reduction in TI activity, whereas soaking them in sodium bicarbonate (pH = 8.4) had no effect on it. Previous studies have indicated that legume TIs exhibit stability across a broad pH range (Benjakul et al. 2000; Chan et al. 2013). In this study, the variations in TI reduction observed in citric acid (CA), water (W), and sodium bicarbonate (SB) solutions likely stem from differences in TI solubility. The solubility of TIs is influenced by factors such as pH and the ionic strength of the soaking medium. Generally, legume proteins exhibit minimal solubility around pH 4–5, which corresponds to their isoelectric point. Carbonaro et al. (1997) reported that solubility significantly increases on either side of these pH values for raw faba beans, lentils, and chickpeas, reaching maximum levels at pH values above 7.0 and below 2.0. The elevated initial pH (8.75) of the SB solution may contribute to increased TI loss due to enhanced solubility. Furthermore, the presence of salts in the SB solution could further modulate TI solubility, affecting the overall reduction trend.

The effect of ultrasound application on TI content varied depending on the type of seed, SS, and ST. In most cases, UAS led to a significant reduction in TI content compared to CS, with reductions ranging from 1.6% to 41.5% in chickpeas, 1.6%–41.6% in beans, and 16.7%–50.1% in soybeans. However, some exceptions were observed, particularly in seeds soaked in SB for 12 h and chickpeas and beans soaked in water for 12 h. The greater loss of TIs during UAS may be partly attributed to the enhanced mass transfer rate generated by ultrasonic waves facilitating the diffusion of soluble TIs from legumes into the soaking medium. Additionally, UAS may cause a loss of structural integrity and inactivation of TIs. Ultrasound treatment affects proteins primarily through physical effects of cavitation, which can disrupt non‐covalent interactions (hydrogen bonds, ionic interactions, hydrophobic interactions) as well as disulfide (S–S) bonds leading to alterations in secondary and tertiary structures (Kozell et al. 2023). Huang et al. (2008) showed that ultrasound at 20 kHz (25°C) significantly altered the secondary conformations of soybean TIs (KTI and BBTI) by reducing disulfide bonds. Structural modifications make TIs more prone to aggregation or solubility changes. For the inactivation mechanism of soybean TIs under low‐frequency ultrasound (20–100 kHz), Wu et al. (2021) proposed that conformational changes and protein denaturation expose previously buried hydrophobic regions, leading to intermolecular aggregation through hydrophobic interactions. Ultrasound typically has minimal effect on covalent peptide bonds, but under extreme conditions of high intensity and prolonged exposure, hydrodynamic shear forces and cavitation may induce peptide bond breakage, leading to protein hydrolysis (Kozell et al. 2023). UAS was consistently more effective than CS across all legumes, with its impact being most pronounced in soybeans. This stronger effect in soybeans may be attributed to differences in the structural composition of TIs. Unlike beans and chickpeas, which primarily contain BBTIs, soybeans contain KTIs and BBTIs. According to Huang et al. (2008), the structural differences between soybean TIs (KTI and BBTI), particularly in their disulfide bonds and secondary conformations, accounted for their varying resistance to ultrasound. Their study demonstrated that after 20 min of ultrasound treatment (20 kHz) in deionized water at 25°C, KTI's disulfide linkages were more easily broken down compared to those of BBTI. Overall, for all seeds, the influence of ultrasound on TI content was less pronounced when sodium bicarbonate was used as a soaking solution. When sodium bicarbonate was used as the soaking solution, the highest difference due to the application of ultrasound was 20% (in soybean samples soaked for 8 h).

### 3.3 Moisture Content of Seeds

The moisture contents of raw chickpea, bean, and soybean were determined as 9.65%, 9.65%, and 9.70% (w.b.), respectively. After soaking treatments, moisture content values reached a value ranging from 47.95% to 54.00% (w.b.) for chickpeas, from 50.29% to 54.60% (w.b.) for beans, and from 52.36% to 57.86% (w.b.) for soybeans (Table 2). The water uptake of legume seeds is a complex process influenced by various factors, including hydration process conditions and intrinsic properties of seeds (e.g., variety, color, size, structure, composition, presence of pores or cracks on the surface of seed coat, initial moisture content) (Miano and Augusto 2018). These factors affect the hydration rate of seeds, their water absorption capacity, and consequently, the moisture values reached at the end of the soaking durations. The higher moisture content of soybean may be attributed to its higher water absorption rate and maximum water holding capacity compared to others. Pan and Tangratanavalee (2003) recommended a minimum moisture content of 120% for soybeans before grinding. In our study, this moisture content was achieved after 4 h of UAS and 8 h of CS.

**TABLE 2:** Moisture content (MC), and hardness values of legume seeds under conventional soaking (CS) and ultrasound‐assisted soaking (UAS).

Soaking treatment	Soaking time (h)	Chickpea		Bean		Soybean	
		MC (w.b. %)	Hardness (N)	MC (w.b. %)	Hardness (N)	MC (w.b. %)	Hardness (N)
CS‐W	4	47.95 ± 0.87b*B**	171.19 ± 23.65aA	50.91 ± 0.93bBC	193.39 ± 24.13aAB	52.36 ± 0.45bC	140.77 ± 18.39aA
	8	50.95 ± 1.22aB	175.24 ± 18.93aA	51.75 ± 0.72bC	200.90 ± 26.02aA	55.75 ± 0.46aAB	135.82 ± 18.28aA
	12	52.19 ± 1.73aA	179.64 ± 20.18aA	53.14 ± 0.73aB	196.85 ± 27.96aA	55.86 ± 0.80aB	136.61 ± 13.60aA
CS‐CA	4	48.34 ± 0.87bB	165.93 ± 22.62aA	50.29 ± 0.93bC	175.51 ± 17.48aB	53.12 ± 0.25cB	139.11 ± 15.78aA
	8	52.01 ± 0.72aAB	166.61 ± 13.98aA	52.26 ± 0.51aBC	187.39 ± 19.34aA	55.47 ± 0.36bAB	129.83 ± 9.62aA
	12	53.26 ± 1.04aA	170.04 ± 23.73aA	52.71 ± 0.89aB	190.81 ± 20.73aA	56.28 ± 0.45aAB	115.58 ± 15.89bB
CS‐SB	4	48.25 ± 0.59bB	179.12 ± 18.06aA	50.87 ± 0.51cBC	205.20 ± 33.62aA	52.58 ± 0.47cBC	137.26 ± 18.63aA
	8	52.08 ± 1.13aAB	176.46 ± 16.01aA	51.79 ± 0.14bC	204.25 ± 35.01aA	55.14 ± 0.29bAB	141.78 ± 15.12aA
	12	52.62 ± 1.04aA	179.05 ± 15.94aA	53.31 ± 0.40aB	195.15 ± 31.63aA	56.34 ± 0.33aAB	135.07 ± 12.53aA
UAS‐W	4	51.98 ± 1.08aA	178.98 ± 19.60aA	52.65 ± 0.43bA	193.91 ± 24.31aAB	54.81 ± 0.23bA	126.45 ± 14.61aA
	8	54.00 ± 1.81aA	180.81 ± 23.20aA	52.97 ± 0.49bAB	214.42 ± 35.27aA	56.12 ± 1.68abA	133.51 ± 11.88aA
	12	53.15 ± 1.23aA	169.40 ± 27.04aA	54.60 ± 0.69aA	188.34 ± 33.10aA	56.89 ± 0.40aAB	136.84 ± 17.82aA
UAS‐CA	4	51.26 ± 1.56aA	166.76 ± 18.73bA	51.68 ± 0.91bAB	193.87 ± 23.53aAB	54.86 ± 0.63bA	132.60 ± 15.70aA
	8	52.67 ± 1.68aAB	172.08 ± 20.95abA	53.00 ± 0.72aAB	189.10 ± 24.74aA	54.40 ± 0.44bB	137.86 ± 14.39aA
	12	53.01 ± 1.24aA	185.63 ± 21.62aA	53.10 ± 0.69aB	206.83 ± 20.24aA	57.86 ± 0.32aA	129.24 ± 16.10aAB
UAS‐SB	4	50.26 ± 0.97bA	168.98 ± 19.03aA	52.22 ± 0.55bA	195.32 ± 19.02bAB	54.80 ± 0.77aA	134.53 ± 17.40aA
	8	52.89 ± 2.23aAB	175.35 ± 14.54aA	53.70 ± 0.45aA	201.82 ± 18.74abA	55.81 ± 1.36aAB	135.29 ± 21.78aA
	12	52.54 ± 1.61abA	177.84 ± 20.15aA	52.42 ± 0.48bB	215.98 ± 28.44aA	56.61 ± 2.05aAB	134.16 ± 18.61aA
**Source**		***p* (*R*** ^**2**^ **= 0.70)**	***p* (*R*** ^**2**^ **= 0.00)**	***p* (*R*** ^**2**^ **= 0.70)**	***p* (*R*** ^**2**^ **= 0.81)**	***p* (*R*** ^**2**^ **= 0.80)**	***p* (*R*** ^**2**^ **= 0.85)**
*SM*		0.000***	0.558	0.000	0.039	0.000	0.532
*SS*		0.528	0.161	0.309	0.003	0.778	0.028
*ST*		0.000	0.216	0.000	0.098	0.000	0.132
*SM × SS*		0.007	0.145	0.968	0.391	0.200	0.089
*SM × ST*		0.000	0.807	0.000	0.427	0.000	0.028
*SS × ST*		0.710	0.432	0.063	0.160	0.003	0.017
*SM × SS× ST*		0.812	0.158	0.031	0.083	0.027	0.436

*Note:* Values are means ± standard deviation of six replicates. *For each soaking treatment, different small case letters in the same column indicates significant difference between soaking times (*P* < 0.05). **For each soaking time, different capital letters in the same column indicates significant difference between soaking treatments (*P* < 0.05). ***p < 0.05 denotes significant effect of main factors or interactions.

Abbreviations: CA, citric acid; SB, sodium bicarbonate; SM, soaking method; SS, soaking solution; ST, soaking time; W, water.

The ANOVA results revealed that the moisture content of all seeds was significantly (*p* < 0.05) influenced by the SM, ST, and the interaction between these two factors (Table 2). During CS of beans, extending ST from 4 to 8 h led to a lower level of increase in moisture content (1.6%–3.9%) compared to the chickpeas and soybeans (4.4%–7.9%). This behavior may be due, at least partially, to a hard seed coat in beans which may hinder hydration during the initial stage of soaking. During soaking, specific microstructural elements like micropyle, hilum, raphe, and seed coat may have possible roles in water entry into pulses. Miano and Augusto (2015) reported that Adzuki beans with moisture content of 14.96% (d.b.) did not hydrate at 35°C when the hilum was covered, which shows the impermeability of the seed coat. In water and sodium bicarbonate‐soaked beans, a rise of 1.6%–1.8% in moisture content was noted with the extension of ST from 4 to 8 h, while a further extension of ST led to a higher (2.7%–2.9%) increase. This situation was different from what was observed in soybeans and chickpeas. This may be at least in part due to the increase in seed coat permeability of beans during soaking. At the beginning of soaking, water entering through the hilum is distributed to the space between the seed coat and cotyledon. Miano and Augusto (2015) stated that the water entering the seed coat‐cotyledon space hydrates the seed coat of beans, leading to an increase in its permeability and subsequently, the seed coat begins allowing water to enter the grain, thereby contributing to the hydration process.

Soaking solution (SS) was not found to be a significant factor (*p* < 0.05) on the moisture content of seeds. Several researchers investigated the influence of the pH of the soaking solution on the hydration rate of legume seeds. Haladjian et al. (2003) showed that the water uptake of faba beans was more rapid and greater in phosphate buffer solutions with alkaline pHs compared to those with acid pHs. In a different study, Vasquez et al. (2021) reported that sodium bicarbonate solution (pH 8.4–8.5) reduced the hydration rate of pigeon pea compared to distilled water (pH 5.7). Contradictory results reveal that the effect of pH on the hydration rate may be influenced by processing factors like seed type, composition of SS, and other processing factors. In this study, it has not been possible to reach a clear conclusion regarding the effect of the pH of SS on the moisture content of seeds.

All seeds subjected to UAS for 4 h had higher moisture content compared to the ones subjected to CS. The SM × SS interaction term was an effective factor for moisture content in chickpeas, but not in beans and soybeans. For chickpeas, the effect of ultrasound was more pronounced when water was used as the SS. Results indicated that the application of ultrasound during soaking accelerated the water absorption rate irrespective of SS. This finding is in accordance with literature (Miano et al. 2019; Kumar et al. 2023; Mazı et al. 2023). Miano et al. (2019) reported approximately an 11% increase in hydration rate for carioca beans ( *P. vulgaris* ) with the application of ultrasound during the soaking process. In their study, ultrasound application reduced the lag phase time and the time required to reach the equilibrium moisture content of beans by approximately 50%. Some researchers have studied the mechanism of ultrasonication during the hydration of pulses and reported that the impact of ultrasonication on the hydration process occurs through two distinct mechanisms: directly and indirectly (Kumar et al. 2023). The direct effect is related to the periodic contractions and expansions of the tissue matrix caused by ultrasonic waves. This is also known as the sponge effect and provides the draw of water into the pores of the pulse by pumping (Patero and Augusto 2015). The indirect effect involves the tissue disruption and formation of microchannels due to acoustic cavitation which improves the mass transfer (Kumar et al. 2023). Kheto et al. (2024a) reported that US pretreatment reduced soaking duration by ~55.55% in guar seeds, attributing this to cavitation‐induced shear forces and collapsing bubbles that weaken cell walls, increase porosity, and create microchannels and nanopores in the outer layer. During UAS, the extension of ST from 4 to 8 h led to a rise in moisture content of chickpeas, beans, and soybeans, but the rate of increase persisted comparably lower than that observed in CS. For chickpeas, the moisture content stayed nearly constant with the additional increase in ST during UAS. Overall, the increase of ST from 4 to 12 h did not create a statistically significant (*p* < 0.05) rise in the moisture content of chickpeas during UAS. This indicates that the application of ultrasound during soaking provided faster attainment of the equilibrium phase in chickpeas. For the beans and soybeans undergoing UAS, the effect of the ST on the moisture content varied depending on the SS. For the beans and soybeans subjected to UAS in water or citric acid, the increase of ST from 4 to 12 h yielded a statistically significant (*p* < 0.05) rise in the moisture content. This may be due, at least partially, to the increase of equilibrium moisture content of these seeds with the application of ultrasound (Miano et al. 2019).

### 3.4 Hardness of Seeds

The hardness values of soaked chickpeas, beans, and soybeans were in the range of 165.9–185.6 N, 175.5–216.0 N, and 115.6–141.8 N, respectively (Table 2). As legume seeds subject to soaking, they absorb moisture, leading to swelling and softening of the seed structure. In this study, increasing ST from 4 to 12 h did not show a statistically significant (*p* < 0.05) effect on the hardness of chickpeas, beans, and soybeans. Reaching the equilibrium hardness value during the initial 4‐h soaking period for chickpeas, beans, and soybeans may be the reason for the minimal changes in hardness after 4 h of soaking. Our findings are in accordance with literature. According to the findings of Chenoll et al. (2009) the hardness of chickpeas, when soaked in water at room temperature, decreased as the moisture level rose during the initial 4‐h soaking period, remaining constant thereafter. They suggested that, during the soaking period of 0 to 4 h, absorbed water is distributed between the starch and protein fractions in chickpea. After 4 h, the chickpea starts to absorb water into the liquid phase only, rather than into the protein or starch phases; thus, this change in water content does not impact the texture. In a different study, de Lima et al. (2014) detected no significant (*p* < 0.05) change in the hardness of soybeans after 1 h of soaking in water at temperatures higher than 25°C. The interaction term of SS × ST had a significant (*p* < 0.05) effect on the hardness for soybeans but not for chickpeas and beans. During CS, the reduction in the hardness of citric acid‐soaked soybeans with the increase of ST from 4 to 12 h was about 17%, while it was 3.0% for water‐soaked samples and 1.6% for sodium bicarbonate‐soaked ones. In the UAS process, the greatest change in hardness with the increase in ST from 4 to 12 h was 11.3% (observed in chickpeas soaked in citric acid solution).

Overall, seeds soaked in citric acid exhibited lower hardness values than those soaked in water and sodium bicarbonate during CS. This trend was not observed in UAS. Statistically, the effect of soaking solution (SS) on hardness was significant (*p* < 0.05) for beans and soybeans but not for chickpeas. During soaking, the hydrolysis of phytates (PA salts) can play a role in the hardness of legume seeds (Zhu et al. 2022). PA, occurring mostly in the cotyledons of legume seeds, can form complexes with positively charged divalent elements such as iron, zinc, magnesium, and calcium, as well as proteins and polysaccharides (Zhang et al. 2022). During soaking, phytase is activated and hydrolyzes phytates, causing the release of Ca^2+^ and Mg^2+^. These minerals migrate to the middle lamella, react with pectic acid, and form insoluble pectate responsible for the hardening of seeds (Chen et al. 2023). In addition, the Ca^2+^ and Mg^2+^ ions released could potentially form crosslinks with the hydrated seed proteins, contributing to their hardness (Wang and Guo 2021). In this study, the PA levels of chickpeas, beans, and soybeans subjected to CS in citric acid were higher compared to the ones subjected to CS in water and sodium bicarbonate at prolonged STs (Table 1). Lower hardness values obtained in citric acid‐soaked seeds may be, at least partially, related to the lower level of phytase‐catalyzed hydrolysis of PA, causing the formation of insoluble pectates in the cell wall. In the UAS process, it is not possible to talk about a clear trend regarding the effect of the solution type on the hardness values of seeds.

The impact of ultrasound application on hardness exhibited variability based on the type of seed, soaking solution (SS), and ST. Statistically speaking, the effect of ultrasound application on hardness appears significant (*p* < 0.05) only for beans. Compared to CS, UAS yielded 0.3%–6.7% higher hardness values for beans and chickpeas soaked in water for 4 or 8 h and a 10.7% higher hardness value for beans soaked in sodium bicarbonate. Apart from these samples, UAS resulted in 0.6%–10.2% lower hardness values compared to CS for the chickpeas, beans, and soybeans soaked in water or sodium bicarbonate. Higher hydration rates and the changes in microstructure of seeds can contribute to the lower hardness values obtained during the UAS process (Li et al. 2019). On the other hand, in citric acid soaking, UAS led to higher hardness values in chickpeas (0.5%–9.2%) and beans (0.9%–8.4%) across all soaking durations and in soybeans (6.2%–11.8%) after 8–12 h of soaking durations compared to CS. During citric acid soaking, the pH increase observed during the UAS process (pH ranged between 4.5 and 5.2) was greater than that seen in the CS process (pH ranged between 3.7 and 4.7). Consequently, it is thought that PA hydrolysis catalyzed by phytase may take place at a higher level in the UAS process as opposed to the CS process, potentially accounting for the rise in hardness values. The samples with the lowest hardness values overall were chickpeas and beans subjected to CS in citric acid for 4 h, and the soybeans subjected to CS in citric acid for 12 h.

### 3.5 Soaking Solution Properties

#### 3.5.1 Soluble Solids Content (SSC)

During the soaking process, water penetrates the seeds, leading to the leaching of soluble substances such as sugars, amino acids, vitamins, pigments, phenolic compounds, organic acids, saponins, and other organic molecules into the soaking medium (Kaur et al. 2016). This influences the SSC of the soaking medium. After soaking treatments, the SSC of water, citric acid, and sodium bicarbonate solutions ranged from 0.2 to 1.52, from 0.40 to 2.33, and from 0.33 to 0.98, respectively (Table 3). The SSC values of the soaking solutions were influenced by the type of seed. However, considering all soaking treatments, it is not possible to make a generalization about the effect of seed type on SSC. For example, the highest SSC values in citric acid solution were obtained when soaking chickpeas, while in sodium bicarbonate solution, they were obtained when soaking soybeans. In water, the situation varied depending on the ST and SM. Njoumi et al. (2019) reported varying amounts of dry matter leached into water during the soaking of different types of legume seeds. They noted a 10% loss of total α‐galacto‐oligosaccharides (raffinose, stachyose and verbascose) in lentils and faba beans and a 40% loss in chickpea seeds after soaking for 16 h in water at 25°C. Some of this loss is caused by leaching into the soaking solution and some is due to enzymatic degradation. Literature studies have shown that the type and amount of soluble substances in seeds and their localization in the seed coat or cotyledon influence the leaching degree.

**TABLE 3:** pH, soluble solids content (SSC), turbidity and color change (Δ*E*) of soaking solutions under conventional soaking (CS) and ultrasound‐assisted soaking (UAS).

Soaking treatment	ST (h)	Chickpea soaking				Bean soaking				Soybean soaking			
		pH	SSC	Turbidity	Δ*E*	pH	SSC	Turbidity	Δ*E*	pH	SSC	Turbidity	Δ*E*
Distilled water		5.95 ± 0.02	0.00 ± 0.00	0.07 ± 0.02	—	5.95 ± 0.02	0.00 ± 0.00	0.07 ± 0.02	—	5.95 ± 0.02	0.00 ± 0.00	0.07 ± 0.02	—
Citric acid		2.57 ± 0.02	0.28 ± 0.04	0.05 ± 0.00	—	2.57 ± 0.02	0.28 ± 0.04	0.05 ± 0.00	—	2.57 ± 0.02	0.28 ± 0.04	0.05 ± 0.00	—
Sodium bicarbonate		8.75 ± 0.03	0.18 ± 0.04	0.05 ± 0.00	—	8.75 ± 0.03	0.18 ± 0.04	0.05 ± 0.00	—	8.75 ± 0.03	0.18 ± 0.04	0.05 ± 0.00	—
CS‐W	4	6.23 ± 0.03a*C**	0.40 ± 0.13bCD	0.04 ± 0.01bB	35.6 ± 1.1bC	6.30 ± 0.04aC	0.30 ± 0.00bE	0.03 ± 0.00bC	36.4 ± 2.3bB	6.34 ± 0.03aC	0.50 ± 0.00bD	0.22 ± 0.05bBC	34.4 ± 0.3cC
	8	5.97 ± 0.01bC	0.20 ± 0.00cE	0.03 ± 0.01bB	53.4 ± 4.1aA	6.00 ± 0.06bC	0.20 ± 0.00cD	0.01 ± 0.00bB	47.6 ± 4.7aB	6.07 ± 0.04bB	0.30 ± 0.00cD	0.19 ± 0.01bD	47.3 ± 1.7aA
	12	6.20 ± 0.02aB	0.65 ± 0.05aE	0.09 ± 0.02aD	34.9 ± 0.4bC	6.24 ± 0.06aD	0.65 ± 0.05aD	0.08 ± 0.05aA	36.3 ± 1.1bE	6.11 ± 0.09bB	0.60 ± 0.00aC	0.41 ± 0.08aD	37.0 ± 1.8bB
CS‐CA	4	3.93 ± 0.01cE	0.77 ± 0.05cB	0.02 ± 0.01bB	38.7 ± 0.4bB	3.71 ± 0.10cF	0.45 ± 0.05bC	0.02 ± 0.01bD	38.6 ± 0.1cA	4.25 ± 0.04cF	0.65 ± 0.05bB	0.13 ± 0.01bD	37.3 ± 0.6cA
	8	4.25 ± 0.01bE	0.90 ± 0.00bC	0.03 ± 0.01bB	41.3 ± 3.5aC	4.13 ± 0.02bE	0.40 ± 0.00bC	0.02 ± 0.01bB	47.4 ± 2.0bB	4.40 ± 0.04bE	0.55 ± 0.05cC	0.13 ± 0.01bE	44.3 ± 1.9bB
	12	4.59 ± 0.01aD	1.12 ± 0.04aC	0.05 ± 0.00aD	37.0 ± 1.1bB	4.63 ± 0.04aF	0.88 ± 0.04aC	0.06 ± 0.01aA	50.3 ± 0.2aB	4.74 ± 0.05aD	1.00 ± 0.00aB	0.19 ± 0.01aE	49.1 ± 0.7aA
CS‐SB	4	7.11 ± 0.02aA	0.33 ± 0.05bD	0.03 ± 0.00cB	31.2 ± 0.2cD	7.14 ± 0.01aA	0.42 ± 0.04bCD	0.03 ± 0.00bC	31.2 ± 0.3cC	7.05 ± 0.02aA	0.60 ± 0.00bBC	0.21 ± 0.00cC	30.0 ± 0.2cE
	8	6.72 ± 0.02bA	0.38 ± 0.08bD	0.07 ± 0.00bB	44.0 ± 2.1aC	6.66 ± 0.02cA	0.35 ± 0.05cC	0.06 ± 0.01aB	40.3 ± 1.8aC	6.70 ± 0.03cA	0.50 ± 0.00cC	0.31 ± 0.01bC	39.6 ± 1.2aD
	12	6.62 ± 0.01cA	0.55 ± 0.05aE	0.25 ± 0.01aB	37.1 ± 2.7bB	6.80 ± 0.01bB	0.70 ± 0.00aD	0.07 ± 0.01aA	38.8 ± 0.7bD	6.78 ± 0.01bA	0.90 ± 0.00aB	0.51 ± 0.02aC	37.7 ± 0.3bB
UAS‐W	4	6.22 ± 0.02cC	0.50 ± 0.00cC	0.08 ± 0.01bA	49.3 ± 1.0aA	6.16 ± 0.03cD	0.35 ± 0.05cDE	0.06 ± 0.01bB	36.2 ± 0.4bB	6.23 ± 0.04aD	0.57 ± 0.05cCD	0.25 ± 0.01cB	35.2 ± 0.7cB
	8	6.35 ± 0.02bB	1.10 ± 0.09bB	0.13 ± 0.02bB	32.1 ± 0.8cD	6.27 ± 0.06bB	0.75 ± 0.05bA	0.07 ± 0.02bB	32.6 ± 0.7cD	5.74 ± 0.19bC	0.85 ± 0.05aA	0.36 ± 0.03bB	41.9 ± 0.8aC
	12	6.67 ± 0.04aA	1.52 ± 0.10aB	0.21 ± 0.07aBC	36.7 ± 1.0bBC	6.94 ± 0.10aA	1.00 ± 0.00aB	0.27 ± 0.08aA	37.2 ± 0.6aE	4.71 ± 0.13cD	0.70 ± 0.11bC	1.01 ± 0.00aA	37.3 ± 1.2bB
UAS‐CA	4	4.46 ± 0.02cD	0.95 ± 0.05cA	0.09 ± 0.02bA	50.4 ± 3.5bA	4.37 ± 0.03bE	0.72 ± 0.04bA	0.16 ± 0.00aA	36.1 ± 0.8cB	4.54 ± 0.05cE	0.83 ± 0.05bA	0.25 ± 0.01bB	34.3 ± 0.4bC
	8	4.86 ± 0.03bD	1.45 ± 0.05bA	0.10 ± 0.02bB	50.1 ± 0.8bB	5.05 ± 0.22aD	0.75 ± 0.05bA	0.05 ± 0.00aB	50.2 ± 0.6bA	4.77 ± 0.07bD	0.80 ± 0.00bA	0.22 ± 0.01bD	44.5 ± 1.1aB
	12	5.16 ± 0.07aC	2.33 ± 0.21aA	0.13 ± 0.01aCD	70.9 ± 0.4aA	5.14 ± 0.05aE	1.38 ± 0.08aA	0.25 ± 0.36aA	72.2 ± 0.7aA	4.89 ± 0.03aC	1.30 ± 0.11aA	0.89 ± 0.04aB	32.9 ± 1.1cC
UAS‐SB	4	7.05 ± 0.04aB	0.38 ± 0.04bD	0.08 ± 0.01cA	34.9 ± 0.8bC	6.91 ± 0.01aB	0.60 ± 0.05bB	0.16 ± 0.00aA	35.5 ± 0.5cB	6.88 ± 0.01aB	0.87 ± 0.05bA	0.30 ± 0.04cA	33.4 ± 0.9aD
	8	6.32 ± 0.45bB	0.52 ± 0.17bD	0.38 ± 0.16bA	44.4 ± 1.2aC	6.51 ± 0.18cA	0.60 ± 0.04bB	0.15 ± 0.09aA	38.9 ± 0.9bC	6.62 ± 0.08bA	0.72 ± 0.08cB	0.46 ± 0.05bA	30.8 ± 0.4cE
	12	4.95 ± 0.50cCD	0.90 ± 0.00aD	0.89 ± 0.12aA	32.2 ± 1.2cD	6.69 ± 0.05bC	0.82 ± 0.06aC	0.20 ± 0.01aA	40.9 ± 0.8aC	4.92 ± 0.02cC	0.98 ± 0.08aB	0.93 ± 0.03aB	32.6 ± 0.4bC
**Source**		***p* (*R*** ^**2**^ **= 0.98)**	***p* (*R*** ^**2**^ **= 0.98)**	***p* (*R*** ^**2**^ **= 0.95)**	***p* (*R*** ^**2**^ **= 0.97)**	***p* (*R*** ^**2**^ **= 0.99)**	***p* (*R*** ^**2**^ **= 0.98)**	***p* (*R*** ^**2**^ **= 0.47)**	***p* (*R*** ^**2**^ **= 0.99)**	***p* (*R*** ^**2**^ **= 0.99)**	***p* (*R*** ^**2**^ **= 0.96)**	***p* (*R*** ^**2**^ **= 0.99)**	***p* (*R*** ^**2**^ **= 0.97)**
*SM*		0.124***	0.000	0.000	0.000	0.000	0.000	0.000	0.000	0.000	0.000	0.000	0.000
*SS*		0.000	0.000	0.000	0.000	0.000	0.000	0.497	0.000	0.000	0.000	0.000	0.000
*ST*		0.003	0.000	0.000	0.000	0.000	0.000	0.000	0.000	0.000	0.000	0.000	0.000
*SM × SS*		0.000	0.000	0.000	0.000	0.000	0.000	0.724	0.000	0.000	0.060	0.000	0.000
*SM × ST*		0.000	0.000	0.000	0.000	0.000	0.000	0.056	0.000	0.000	0.000	0.000	0.000
*SS × ST*		0.000	0.000	0.000	0.000	0.000	0.000	0.212	0.000	0.000	0.000	0.000	0.000
*SM×SS×ST*		0.000	0.000	0.000	0.000	0.000	0.000	0.458	0.000	0.000	0.000	0.000	0.000

*Note:* Values are means ± standard deviation of six replicates. *For each soaking treatment, different small case letters in the same column indicates significant difference between soaking times (*p* < 0.05). **For each soaking time, different capital letters in the same column indicates significant difference between soaking treatments (*p* < 0.05).***p < 0.05 denotes significant effect of main factors or interactions.

Abbreviations: CA, citric acid; SB, sodium bicarbonate; SM, soaking method; SS, soaking solution; ST, soaking time; W, water.

Under similar soaking conditions, citric acid solution had higher SSC values compared to other soaking solutions, particularly in the soaking of chickpeas. This may be partly due to the enhanced solubility and leaching of certain compounds like calcium or magnesium phytate salts (Amat et al. 2023) under acidic conditions. Additionally, the reduced hardness of seeds observed during citric acid soaking may facilitate the extraction of certain compounds, resulting in higher SSC values. The literature reveals that the acidity of the soaking solution can differentially affect the leaching of various compounds found in legume seeds (Frias et al. 2000; Prodanov et al. 2004). ST was a significant (*p* < 0.05) factor on SSC of soaking solutions. A linear increase (*R* ^2^ > 0.9) over time was observed in the SSC of citric acid and sodium bicarbonate solutions during chickpea soaking. Similarly, SSC of water increased linearly with ST during UAS of chickpeas and beans. In other soaking solutions, the SSC of solutions remained constant or slightly decreased between 4 and 8 h of soaking, followed by a significant (*p* < 0.05) increase from 8 to 12 h. The slight reductions in SSC during soaking treatments may be due to the reabsorption of some solubilized substances back into the seeds, which is caused by the change in the concentration gradient between the soaking solution and the seed interior as soaking progresses. 12 h‐soaking treatments resulted in the highest SSC values for all samples. The trend of SSC changes over time was similar between the UAS and CS methods for citric acid and sodium bicarbonate solutions, but not for water. UAS caused higher SSC values compared to CS. This may be due to the increased mass transfer rate during UAS, which accelerates the diffusion of solutes to the soaking solution. Tissue disruption and formation of microchannels due to acoustic cavitation may enhance the extraction of some compounds. Yıldırım and Öner (2015) noted that the application of ultrasound during soaking of chickpea in water at 87°C–97°C promoted the leaching of electrolytes and soluble solids. The highest effect of ultrasound application on SSC was observed during 8 h‐water soaking treatments, resulting in 3–6 times higher SSC values compared to CS. Moreover, some researchers reported that ultrasound treatment increased the solubility of proteins in pulses (Kumar et al. 2023).

#### 3.5.2 pH

The initial pH of distilled water, citric acid solution, and sodium bicarbonate solution was 5.95, 2.57, and 8.75, respectively (Table 3). During the soaking of legume seeds, various soluble substances leaching into the soaking solution may lead to alterations in the pH levels (Njoumi et al. 2019). Following CS treatments, the pH of water, citric acid, and sodium bicarbonate solutions ranged between 5.97–6.34, 3.71–4.74, and 6.62–7.14, respectively. Following UAS treatments, the pH of water, citric acid, and sodium bicarbonate solutions ranged between 4.71–6.94, 4.37–5.16, and 4.92–7.05, respectively. The main factors of SM, ST, and their interactions were all found to be effective factors in pH values of soaking solutions (Table 3). For all seeds, the variation in the pH of soaking solutions was higher during UAS compared to CS. This may be attributed to the increased level of leached substances into the soaking solution with the application of ultrasound. UAS yielded a higher increase in pH of citric acid solution and a higher reduction in pH of sodium bicarbonate solution. The effect of the ultrasound application on the pH of the water varied according to ST and the seed type. Overall, the most notable effect of ultrasound on pH was observed during 12 h‐soaking of soybeans or chickpeas in sodium bicarbonate and during 12 h‐soaking of soybeans in water.

#### 3.5.3 Turbidity

After soaking treatments, the turbidity values of water, citric acid, and sodium bicarbonate solutions ranged from 0.01 to 1.01, from 0.02 to 0.89, and from 0.03 to 0.93, respectively (Table 3). Turbidity can result from compound leaching into the soaking solution. Considering all soaking treatments, soybeans caused higher turbidity values compared to chickpeas and beans. ST was a significant (*p* < 0.05) factor in the turbidity of soaking solutions. The observed trend indicates that the increase in turbidity is more pronounced when extending the duration from 8 to 12 h compared to the increase observed when extending it from 4 to 8 h. Soaking solution was found to be a significant (*p* < 0.05) factor for chickpeas and soybeans but not for beans. In chickpea soaking, water and citric acid exhibited similar turbidity across all soaking treatments, while sodium bicarbonate had higher turbidity at extended STs. In soybean soaking, the lowest turbidity values were generally observed in the citric acid solution. In bean soaking, a clear comparison between the turbidity values of soaking solutions was not feasible. For all treatments, UAS caused higher turbidity values compared to CS. This result is consistent with the higher SSC values obtained in the UAS treatments. The degree of the effect of ultrasound application on turbidity values varied depending on the seed type and ST. In the case of soybeans, the most notable effect of ultrasound application was observed after 12 h soaking treatments, irrespective of the type of solution used. For chickpeas, the most pronounced effect occurred after 12 h soaking in sodium bicarbonate solution. For beans, the turbidity‐increasing effect of ultrasound remained considerably lower compared to other seeds.

#### 3.5.4 Color Change

Color change (Δ*E*) of soaking solutions ranged between 31 and 71 during chickpea soaking, between 31 and 72 during bean soaking, and between 30 and 49 during soybean soaking (Table 3). The variation in the type and amount of the leached compounds influences the color of soaking solutions. During soaking of chickpeas in water at high temperatures (87°C–90°C) for 260 min, Yıldırım and Öner (2015) recorded an increase in L*, a*, and b* values of water and attributed this to the leaching of starch, yellow color pigments, vitamins, and destruction of carotenoids. The seed coat color of chickpeas, soybeans, and beans is closely correlated to their phenolic compounds, particularly flavonoids, tannins, and phenolic acids (Singh et al. 2017). The legume seeds used in this study are within less pigmented varieties, which tend to contain lower levels of these compounds. Some of these compounds are water soluble and leach into the soaking solution during the soaking process (Singh et al. 2017). In addition to the amount of pigment that passes into the SS, the stability of the pigment in the SS can also influence the color of SS. It is known that the stability of pigments depends on pH (Tanaka et al. 2008). Δ*E* of soaking solutions followed a steady increase with increasing ST only in some samples. Reductions in Δ*E* were observed at prolonged STs for some samples, which may be, at least partially, due to the reabsorption of some compounds. Δ*E* values detected in citric acid solutions were higher compared to those of sodium bicarbonate solutions. This may be related to the higher amount of soluble solids leaching during citric acid‐soaking treatments. For all seeds, the lowest color change was obtained in sodium bicarbonate solutions following 4 h‐ CS treatments. The effect of ultrasound application on color change was variable. The most obvious effect of ultrasound application was observed in citric acid solutions following 12 h soaking treatments. 12 h UAS soaking produced higher color change in citric acid during chickpea and bean soaking, while it gave lower color change in citric acid during soybean soaking.

#### 3.5.5 Multivariate Analysis

Pearson correlation analysis was conducted to examine the bivariate relationships among key variables, including moisture content, hardness, trypsin inhibitor (TI), PA, and pH, with separate assessments for chickpea, bean, and soybean samples (Table 4). The analysis revealed several key relationships among these measured variables. All three legumes exhibited a strong negative relationship between moisture content and anti‐nutritional factors, indicating that as legume seeds absorbed more water, anti‐nutritional factors decreased. Additionally, PA and TI were also significantly correlated, reinforcing their tendency to behave similarly during soaking. pH showed a moderate positive correlation with hardness (0.559, *p* < 0.05) in beans but was not strongly correlated with key variables in chickpeas and soybeans. Although analysis of variance (ANOVA) indicated that soaking solution significantly (*p* < 0.05) influenced TI content, Pearson correlation analysis did not show a correlation between pH and TI content. The reason may be that Pearson correlation could fail to detect this relationship if it is not strictly linear or if it depends on the influence of another variable.

**TABLE 4:** Pearson correlation coefficients among different variables for chickpea (C), bean (B), and soybean (S).

	Moisture content	Hardness	Trypsin inhibitor	Phytic acid
Hardness	C: 0.331 B: 0.168 S: −0.419			
Trypsin inhibitor	C: −0.715** B: −0.664** S: −0.805**	C: −0.365 B: −0.248 S: 0.169		
Phytic acid	C: −0.549* B: −0.757** S: −0.653**	C: −0.431 B: −0.369 S: 0.038	C: 0.694** B: 0.746** S: 0.563*	
pH	C: −0.048 B: 0.296 S: −0.240	C: 0.432 B: 0.559* S: 0.317	C: 0.051 B: −0.098 S: 0.053	C: 0.004 B: −0.323 S: 0.043

*Note:* Significance level: *p < 0.05; **p < 0.01.

**FIGURE 2:**
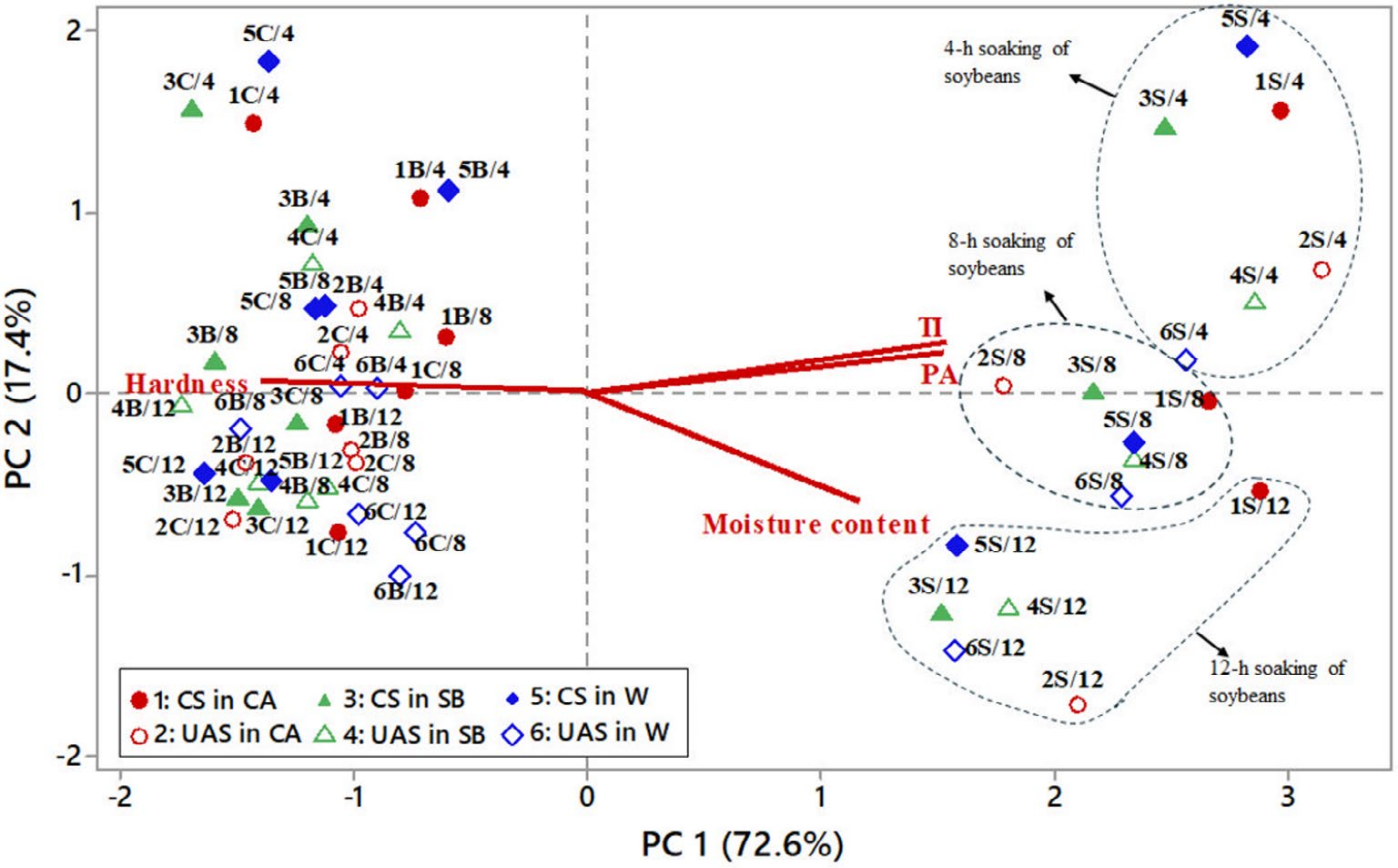
PCA biplot representing the variation in legume seed properties (moisture content, hardness, phytic acid (PA), and trypsin inhibitor (TI)) across different treatments. Data points represent different soaking treatments, where the first number (1–6) indicates the soaking condition given in the legend, the letters B, C, and S refer to bean, chickpea, and soybean, respectively, and the last number (4, 8, or 12) corresponds to the soaking time (h). CS: conventional soaking, UAS: ultrasound‐assisted soaking, CA: citric acid, SB: sodium bicarbonate, W: water.

Principal Component Analysis (PCA) was conducted to explore the underlying patterns in the dataset and assess how soaking treatments influenced the physicochemical properties of legume seeds. Four principal components (PCs) were derived, explaining different proportions of variance in the dataset (Table 5). PC1 (72.6%) explained the majority of variance, indicating that it is the most influential component in differentiating the samples. PC2 (17.4%) contributed additional variation, bringing the total to 90%, meaning the first two PCs account for nearly all of the variance. TIs (0.533) and PA (0.527) were positively correlated with PC1, indicating that samples with higher anti‐nutrient content were positioned along PC1. Hardness had a strong negative loading (−0.526) on PC1, meaning that samples with greater hardness tended to have lower PC1 scores and were positioned to the left. Moisture content (−0.852) strongly influenced PC2 in a negative direction, meaning that samples with higher moisture had lower PC2 values. The PCA biplot (Figure 2) visualizes how different soaking treatments affected the physicochemical properties of legume seeds. In the PCA biplot, soybean samples were strongly separated from chickpea and bean samples. Soybean samples were positioned on the right side (high PC1 values), while chickpea and bean samples were clustered on the left. Soybeans differed from chickpeas and beans primarily due to higher TI, PA, and moisture content, and lower hardness. Soybean samples were more widely spread along PC2, indicating that soaking treatments introduced more variability in soybeans compared to chickpeas and beans. By examining the PCA biplot, it was evident that samples soaked for longer durations tended to separate along PC2, with 12‐h soaked samples positioned lower, indicating higher moisture absorption. This suggests that ST significantly influenced the factor represented by PC2, which, based on the loadings, was moisture content (−0.852 loading on PC2). This trend was particularly evident in soybean samples, which exhibited a clear decrease in PC2 values as ST increased, as shown in Figure 2. Soybean samples soaked for longer durations tended to have lower PC1 values, suggesting that extended soaking helped reduce anti‐nutritional factors. However, a similar trend could not be observed for beans and chickpeas in the PCA biplot. The difference between samples subjected to CS and UAS was also noticeable, with UAS‐treated samples generally exhibiting lower PC2 values than CS‐treated samples. However, this trend was not universal across all STs and legume types. The effect of UAS was more pronounced in the 4‐h ST for all seeds, suggesting a noticeable increase in moisture absorption in the early stages of soaking. Soybeans showed the most prominent separation between UAS and CS, while for chickpeas and beans soaked for 12 h, some UAS‐treated samples overlapped or even had slightly higher PC2 values than CS‐treated samples. TI and PA had strong positive loadings on PC1, meaning that a decrease in PC1 values would indicate a reduction in these compounds. However, we could not observe a clear differentiation between CS and UAS‐treated samples along PC1 in the PCA biplot.

**TABLE 5:** Principal component loadings and variance contributions for legume seed properties.

Variable	PC1	PC2	PC3	PC4
Moisture content	0.401	−0.852	0.295	−0.160
Hardness	−0.526	0.083	0.790	−0.303
Trypsin inhibitor	0.533	0.400	0.027	−0.745
Phytic acid	0.527	0.326	0.537	0.573
Variance (%)	72.6	17.4	7.2	2.8

**FIGURE 3:**
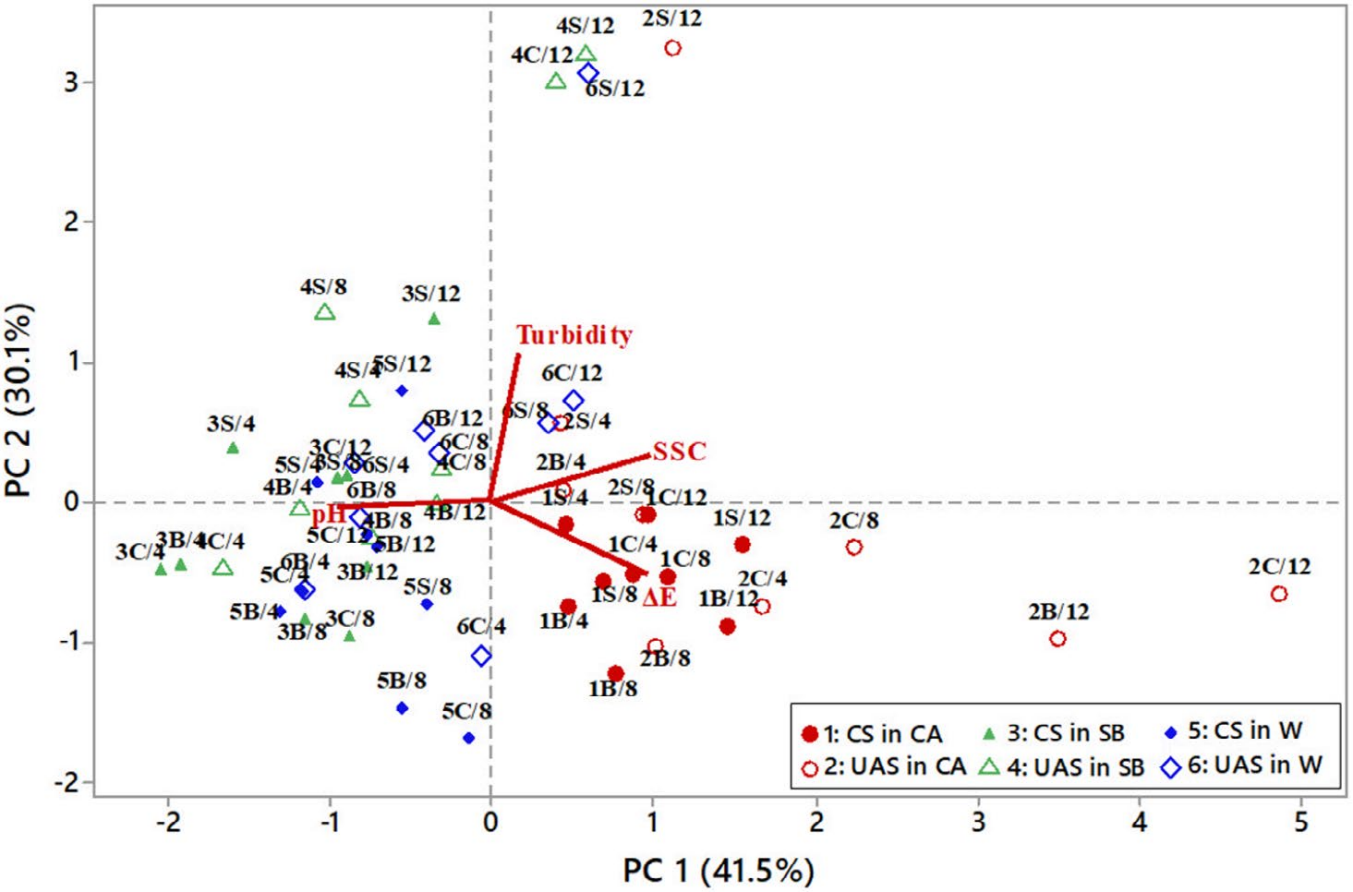
PCA biplot representing the variation in soaking solution properties (pH, soluble solids content (SSC), turbidity, and color change (ΔE)) across different treatments. Data points represent different soaking treatments, where the first number (1–6) indicates the soaking condition given in the legend, the letters B, C, and S refer to bean, chickpea, and soybean, respectively, and the last number (4, 8, or 12) corresponds to the soaking time (h). CS: conventional soaking, UAS: ultrasound‐assisted soaking, CA: citric acid, SB: sodium bicarbonate, W: water.

PCA was also conducted to evaluate the impact of soaking treatments on the properties of the soaking solutions. Four PCs were derived, explaining different proportions of variance in the dataset (Table 6). PC1 accounts for the highest variance (41.5%), followed by PC2 (30.1%), PC3 (18.1%), and PC4 (10.3%). Together, PC1 and PC2 explain 71.6% of the total variance, meaning they capture most of the dataset's patterns. PC1 is highly influenced by SSC (0.597) and color change (0.578), while pH (−0.550) is negatively correlated (Table 6). This suggests that soaking solutions with low pH tend to have higher SSC and color change. PC2 is dominated by turbidity (0.856), meaning this component captures variations related to solution clarity. Turbidity and pH are nearly orthogonal (90°) in the loading plot, meaning these two variables are not likely to be correlated. The PCA biplot (Figure 3) visualizes how different soaking treatments group based on their principal components (PC1 & PC2). Each data point in the PCA score plot represents a soaking solution with a specific soaking treatment. Citric acid (CA) soaking solutions were clustered on the right side of the plot, with those subjected to longer STs positioned further to the right. In contrast, sodium bicarbonate (SB) and water (W) soaking solutions were primarily clustered on the left side, except for some samples that underwent UAS for extended durations. SB and W soaking solutions subjected to UAS for 12 h were positioned higher on PC2, indicating that longer UAS treatments had a greater impact on solution property changes. Notably, UAS‐treated CA samples were more widely spread, suggesting that UAS had a stronger modifying effect on solution properties in CA compared to SB and W. Overall, UAS‐treated soaking solutions were more dispersed than conventionally soaked ones, with the extent of dispersion influenced by both ST and the soaking medium. This trend highlights that longer UAS treatments introduced greater variation in solution properties, particularly in CA solution.

**TABLE 6:** Principal component loadings and variance contributions for soaking solution properties.

Variable	PC1	PC2	PC3	PC4
pH	−0.550	−0.033	0.819	0.162
Soluble solids content	0.597	0.272	0.520	−0.510
Turbidity	0.085	0.856	−0.009	0.510
Color change	0.578	−0.439	0.243	0.644
Variance (%)	41.5	30.1	18.1	10.3

## 4 Conclusions

This study demonstrated that soaking treatments effectively reduced PA and TIs in chickpeas, beans, and soybeans, with their efficiency varying based on ultrasound application, soaking solution, and duration. UAS generally improved TI reduction but had a limited effect on PA content. Among the soaking solutions, sodium bicarbonate proved to be the most effective for TI reduction. ST played a crucial role in decreasing both PA and TIs, with longer durations leading to greater reductions. UAS also accelerated water absorption across all seed types but had varied effects on hardness compared to CS. Citric acid‐soaked samples generally exhibited lower hardness during CS. The properties of the soaking solutions, including soluble solids content (SSC), pH, turbidity, and color change, were notably influenced by the soaking conditions and ultrasound application. UAS treatments led to higher SSC, greater pH fluctuations, and increased turbidity, highlighting its potential to accelerate mass transfer processes. Citric acid solutions showed higher SSC values, particularly for chickpeas, which may be attributed to enhanced solubility and leaching of specific compounds under acidic conditions.

These findings offer valuable insights into optimizing legume soaking processes for industrial applications, especially where faster hydration and effective anti‐nutrient reduction are desirable. Future research could explore the combined effect of UAS and pH variations on the structural modifications of TIs to better understand the underlying mechanisms of TI reduction. Additionally, investigating how different soaking treatments influence nutritional quality and protein functionality would provide a more comprehensive understanding of their practical applications in food processing.

## Author Contributions

**Bekir Gökçen Mazı:** conceptualization (lead), data curation (lead), formal analysis (lead), methodology (lead), supervision (lead), writing – original draft (lead). **Kübra Çağlayan:** data curation (supporting), formal analysis (supporting).

## Ethics Statement

The authors have nothing to report.

## Consent

Written informed consent was obtained from all study participants.

## Conflicts of Interest

The authors declare no conflicts of interest.

## Data Availability

The datasets used and/or analyzed during the current study are available from the corresponding author on reasonable request.
